# Down-regulation of MSMO1 promotes the development and progression of pancreatic cancer

**DOI:** 10.7150/jca.73112

**Published:** 2022-08-01

**Authors:** Rongxian Cao, Zhiqiang Zhang, Chen Tian, WeiWei Sheng, Qi Dong, Ming Dong

**Affiliations:** 1 Department of Gastrointestinal Surgery, The First Hospital of China Medical University, China.; 2 Department of General Surgery, The People's Hospital of Liaoning Province, Shenyang, China.

**Keywords:** MSMO1, Pancreatic Cancer, EMT, PI3K-AKT-mTOR, Progression

## Abstract

**Background:** Methylsterol monooxygenase 1 (MSMO1), as a completely unique tumor biomarker, plays a vital role in the malignant progression of various cancer. Until now, the potential function and pathway of MSMO1 in the development of pancreatic cancer (PC) has not been explored yet, to our knowledge.

**Methods:** We systematically explored the detail function of MSMO1 in Epithelial-mesenchymal transition (EMT) and cell proliferation of PC *in vitro* and *in vivo*.

**Results:** MSMO1 expression was much lower in PC tissues than that in paired normal pancreas. MSMO1 positive expression was negatively associated with T stage, lymph node metastasis and vascular permeation of PC patients. Meanwhile, positive MSMO1 expression indicated a significantly better prognosis and an independent favorable prognostic factor. MSMO1 silencing promoted cell invasion and migration via activating EMT and PI3K-AKT-mTOR pathway [p-PI3K (Tyr458), p-AKT (Ser473) and p-mTOR (Ser2448)] in Capan-2, Panc-1 and SW1990 cells. *In vivo*, subcutaneous tumor size was enhanced by MSMO1 silencing following with the consistent change of EMT and PI3K/AKT signaling shown *in vitro*. The motivation of EMT and PI3K-AKT-mTOR pathway was also demonstrated in MSMO1 silencing mouse PANC02 cells.

**Conclusion:** Down-regulation of MSMO1 in PC was associated with advanced progression and poor prognosis of PC patients. MSMO1 acts as a tumor suppressor via inhibiting the aggressive malignant biology of PC accompanying with regulating EMT and PI3K/AKT signaling.

## Introduction

Pancreatic cancer (PC) is one of the most lethal tumors that attributes to aggressive malignant biological behavior 1. The 5-year survival rate of PC keeps going down and the incidence rate remains continuously rising 2. Until 2030, PC is forecast for the second leading cause of death associated with cancer in the world 3. It is urgent to discover the new biomarkers for targeting the malignant biology for PC patients. Epithelial-mesenchymal transition (EMT) is a cellular process initiated from cellular micro environment following the acquisition of mesenchymal phenotypes and the repression of epithelial features, which finally contributes to the cell invasion and metastasis of cancer, including PC 4, 5. The key characteristic of EMT is the deficiency of epithelial markers, such as E-cadherin and the gain of interstitial markers, such as Vimentin and MMPs 6. Increasing evidence confirms the significant role of PI3K-AKT-mTOR signaling pathway and it's downstream targets (p-AKT, p-PI3K and p-mTOR) in regulating EMT process 7, 8. Methylsterol monooxygenase 1 (MSMO1), as a synonym for sterol-C4-methyl oxidase analog (SC4MOL), displays critical function in the normal synthesis of cholesterol 9. Recently, MSMO1 has been reported to promote the development of various cancers including liver 10, breast 11, and oligodendroglioma 12. However, the mechanism and function of MSMO1, especially in PC, is still unknown, to our knowledge. In current study, we systematically investigated the potential role and mechanism of MSMO1 in the progression of PC *in vitro* and vivo.

## Materials and methods

### Cell Lines and Culture

Capan-2, Panc-1, and SW1990 cell lines were offered by General Surgery Laboratory of China Medical University (Shenyang, China) and Panc02 cell lines was obtained from Cell Biology Institute of China Medical University. All cell lines were cultured in temperate situation according to the recommendation from ATCC, such as suitable culture medium within one percent penicillin-streptomycin combination (HanHeng, China) added ten percent fetal calf serum (Gibco Invitrogen, Carlsbad, CA) and maintained in 37 °C humidified incubators filled with five percent CO_2_. All cell lines were detected for mycoplasma free before experiments.

### Tissue Samples

92 pairs of fresh pancreatic cancer samples and paired adjacent normal pancreatic tissues were collected from surgical treatment patients at the Gastrointestinal Surgery Department of the First Hospital of China Medical University and the General Surgery Department of the People's Hospital of Liaoning Province between 2010/9 and 2020/12. All the patients were with complete follow up data and pathologically diagnosed as PC. 8^th^ AJCC Cancer Staging Manual was utilized to classify staging standard. Meanwhile, 22 pairs of PC fresh samples were collected under -80 °C condition for qRT-PCR assays.

### Experimental Animals

All C57BL/6 mice used in this study, weighting 20 to 25 g, were purchased from the company of Beijing Vital River Laboratory Animal Technology (Beijing, China). The animals were kept in the specific pathogens free (SPF) Animal Experimental Ministry of China Medical University with standard conditions (temperature 24±2 °C, humidity 55±10%, and 12-hour day/night cycle). Free access to rodent chow and water were applied for experimental animals. All animal experiments were performed strictly according to institutional regulations in facilities approved by the Animal Care Committee of China Medical University in accordance with Chinese government guidelines for animal experiments.

### Immunohistochemistry

Paraffin embedded PC tissues fixed by neutral formaldehyde. Tissues were used to cut 4 µm slices and dewaxing. Antigen retrieval was next done for 3 minutes under high pressure. Three percent H_2_O_2_ and goat non-immune serum were utilized to block tissues for 30 minutes. Primary antibody incubated overnight with at 4 °C. Sections were incubated with the secondary antibody for 30 minutes at room temperature and then observed color reaction via using 3,3′ -diaminobenzidine (DAB). Immunohistochemistry (IHC) score was conducted as previously described 13. Four grades: [0 (negative), 1 (weak), 2 (medium), and 3 (strong)] was described the positive staining intensity. The 5 grades: [0 (0%), 1 (1-25%), 2 (26-50%), 3 (51-75%), and 4 (76-100%)] were used to calculate according to the stained positive areas. The final scores were calculated by three professional pathologists. The final score, ranging 0-12, was the multiplication of two grades and >6 was regarded as high expression.

### Hematoxylin-Eosin Staining

Briefly, xylene I and II each for 10 minutes was used to dewax the slices. 100%, 90%, 80%, 70% alcohol for 5 minutes each, hematoxylin for 5 minutes, 5% acetic acid differentiation for 1 minute, tap water for 1 minute, eosin for 3 minutes, 70%, 80%, 90%, 100% alcohol each for 10 seconds, xylene I and II for 5 minutes.

### EMT Construction

We transfected Capan-2, PANC-1 and SW1990 cell lines with negative control and MSMO1 siRNA. Recommended growth media (RPMI 1640 for Capan-2 and SW1990 cells, DMEM for Panc-1 cells) containing 1% FBS was used to better induce EMT phenotype in transfected cell lines. The EMT construction model was confirmed by the alteration involving EMT-like cell morphology (a spindle-shaped and fibroblast-like morphology), EMT enhanced cell motility, and the changes of EMT-related markers, which were regarded to reflect the EMT process.

### Western Blot

RIPA lysis buffer containing 1% PMSF (Beyotime, China) was used to collect total protein from cell lines. 10% SDS-PAGE was used to separate protein and PVDF membranes were loaded with separate protein. All membranes were blocked with 5% degreasing milk and incubated with primary antibodies: MSMO1 (Abcam), E-cadherin (Abcam), β-catenin (Proteintech), Vimentin (Proteintech), AKT (Bimake), PI3K (Bimake), m-TOR (Bimake), p-AKT (Ser473) (Cell Signaling Technology), p-PI3K (Tyr458) (Cell Signaling Technology), p-mTOR (Ser2448) (Cell Signaling Technology), GAPDH (Proteintech) overnight at 4 °C. Next day, membranes were incubated with secondary antibodies for two hours. Finally, blots were detected by ECL kit (Beyotime, China). The experiments were done thrice.

### Real-time Quantitative PCR

TRIZOL reagent (Takara, Japan) was used to extract total RNA from tissues samples, following the instructions. RNA level was maintained to the same level in each sample by using nucleotide test. GenePharma (Soochow, China) provided the primers for qRT-PCR. The primer sequences were manifested in **Table 1**. The total RNA and TaKaRa RNAiso Reagent (Takara, Japan) was used to synthesize cDNA. Finally, the QuantStudio 7 Pro Real-Time PCR System (Thermo Fisher Scientific, USA) was used to measure the mRNA expression of MSMO1 and GAPDH under the conditions as below: 95 °C for 30s, 45 cycles of 95 °C for 5s and 60 °C for 45s. The -ΔΔCt method with melt-curve dissociation was used to evaluate the quality of amplification products. The experiments were repeated three times.

### RNA Interference

The MSMO1 siRNA and negative control (NC) were designed by OriGene (OriGene Technologies, Inc. USA). The verified sequence (OPF) of MSMO1 siRNA were as followed: Sense: GAAGCCCUUUAUUUUCUUAUTT. Anti-sense: AUAAGAAAUAAAGGGCUUCTT. Capan-2, Panc-1, and SW1990 cells were transiently transfected with MSMO1 siRNA and NC for 24 hours with lipo3000 (Invitrogen, Carlsbad, CA, USA) following the instruction. In the result section, the silencing effect of MSMO1 was testified.

### Cell Migration and Invasion Assays

Based on our previous study 14, for cell migration assay, 2×10^5^ cells/ml of Capan-2 cells, 3*10^5 cells/ml of Panc-1 cells and 1.5×10^6^ cells/ml of SW1990 cells were implanted into chamber inserts with serum-free growth media in 24-well plates within growth media containing 10% FBS at the bottom of each well as a stimulus after transfected for 24 hours. 24 hours later, the per-cooling methanol was used to fix the migrated cells and the Crystal Violet (Sigma) was used for dyeing. The invasion assay was similar to migration that the top of membrane coated with Matrigel (BD Biosciences, USA). A microscope (Nikon Microphot-FX, Japan) was used to calculate the final migrating and invading cells at 20× magnification in five random fields each chamber. Each independent experiment was performed thrice.

### *In vivo* Cell-line-Derived Tumor Xenograft

To detect the function of MSMO1 in cell proliferation, total six C57B6 mice were used to construct subcutaneous tumor model by injecting PANC-02 cells resuspended in PBS which were transfected with MSMO1 siRNA and negative control respectively (1×10^6^/ml, 100 ul). The selected mice are generally 4 weeks old and the middle of the armpit was chosen as the planting site. After injection, cotton swab was used to reduce bleeding and the overflow of the cell suspension from the injection site. Four weeks later, the mice were sacrificed and tumors were resected. The longest and shortest parts of the tumor are measured using a vernier caliper with the following formula: V=1/2*a*b^2^ (a is the long axis, b is the short axis). After the subcutaneous tumor is removed, a part of tumors was used for later extraction of protein and other was used for hematoxylin and eosin (HE) and IHC staining.

### Statistical Analysis

Statistical analysis was emerged from SPSS 20.0 software (Chicago, IL, USA). Three independent experiments results were presented by mean ± SD. Discrepancises between two groups were compared through independent t test. MSMO1 and clinicopathological parameters were compared by chi-square test. To deal with survival curve, Kaplan-Meier method was utilized and significance was compared via log-rank test. Independent prognostic factors were evaluated by Cox's proportional hazards regression model. P<0.05 was considered as statistically significant.

## Results

### MSMO1 is Down-Regulated in PC and Associated with The Aggressive Clinical Stage and Prognosis of PC Patients

**Figure 1:**
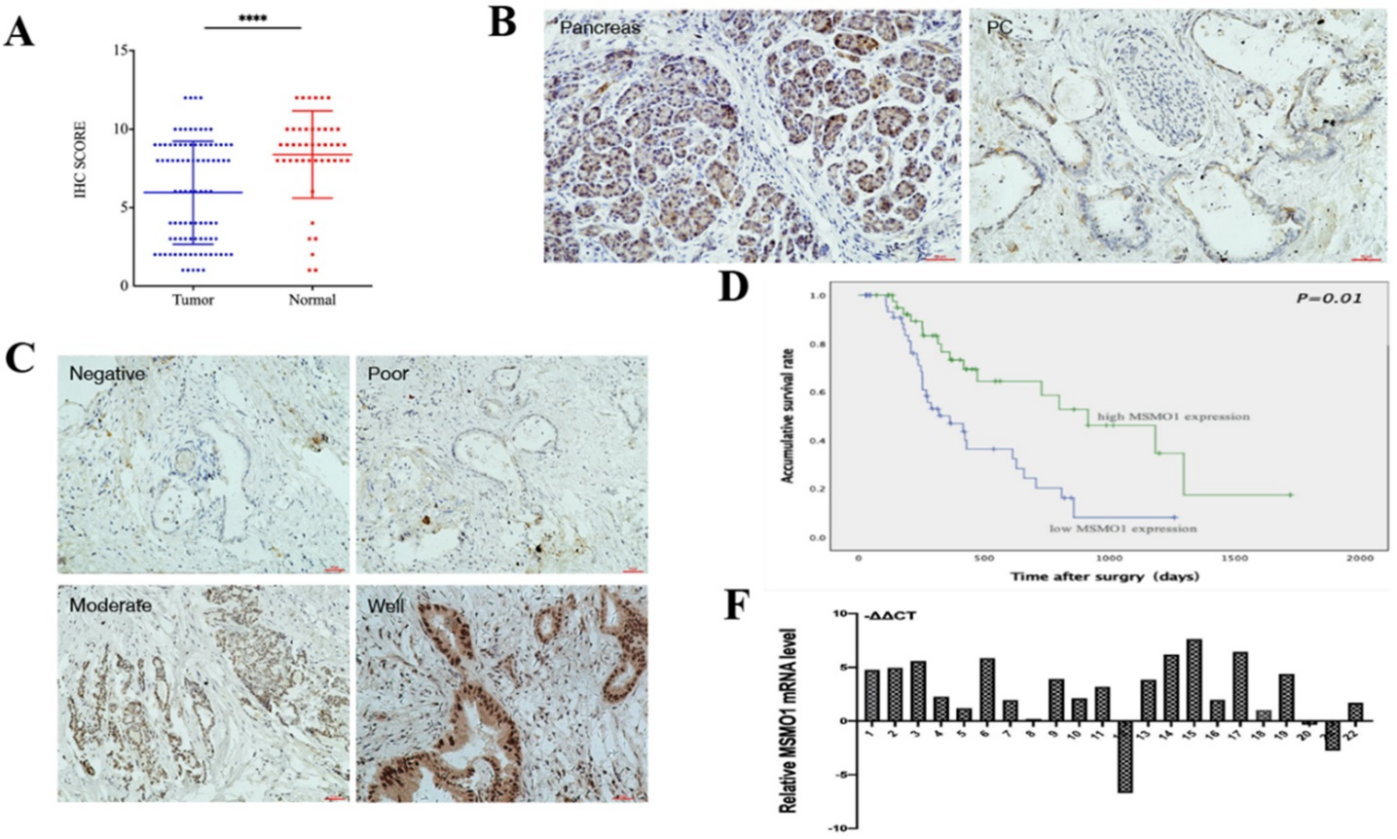
** The MSMO1 expression in PC tissues. (A)** Immunohistochemistry was used to calculate the expression of MSMO1 in the cancer tissues and normal tissues. **(B)** MSMO1 expression in PC tissues and normal pancreatic tissues using IHC. **(C)** MSMO1 expression intensity in different Differentiation levels of PC tissues. **(D)** Kaplan-Meier survival curves in PC patients with different MSMO1 expression. **(F)** MSMO1 mRNA expression quantities in 22 pairs tissue samples via qRT-PCR. ****P < 0.0001 compared with the control.

In our study, IHC showed MSMO1 expression in human PC tissues (43/92,46%) was lower compared with normal tissues (38/45, 84%) (*P*<0.0001) **(Figure 1A)**. MSMO1 was mainly expressed in the cytoplasm with a small amount of nuclear expression in human PC tissues **(Figure 1B)**. Meanwhile, As the degree of tumor differentiation enhancing, the expression of MSMO1 increases **(Figure 1C)**. In current study, high MSMO1 expression was negatively relevant with T stage (P=0.025), lymph-node metastasis (P=0.046) and vascular permeation (P=0.009) of 92 PC patients **(Table 2)**, patients with low MSMO1 expression had a poor prognosis shown by Kaplan-Meier curve (P=0.01) **(Figure 1D)**. In addition, the low MSMO1 expression independently causes poor prognosis was observed by univariate and multivariate Cox's regression analysis results **(Table 3)**. Compared with the PC tissues, higher mRNA expression of MSMO1 in normal pancreatic tissues were shown by qRT-PCR assay (P=0.009) **(Figure 1F)**.

### MSMO1 Inhibits Epithelial-Mesenchymal Transition, The Capacities of Cell Malignant Migration and Invasion *in vitro*

**Figure 2:**
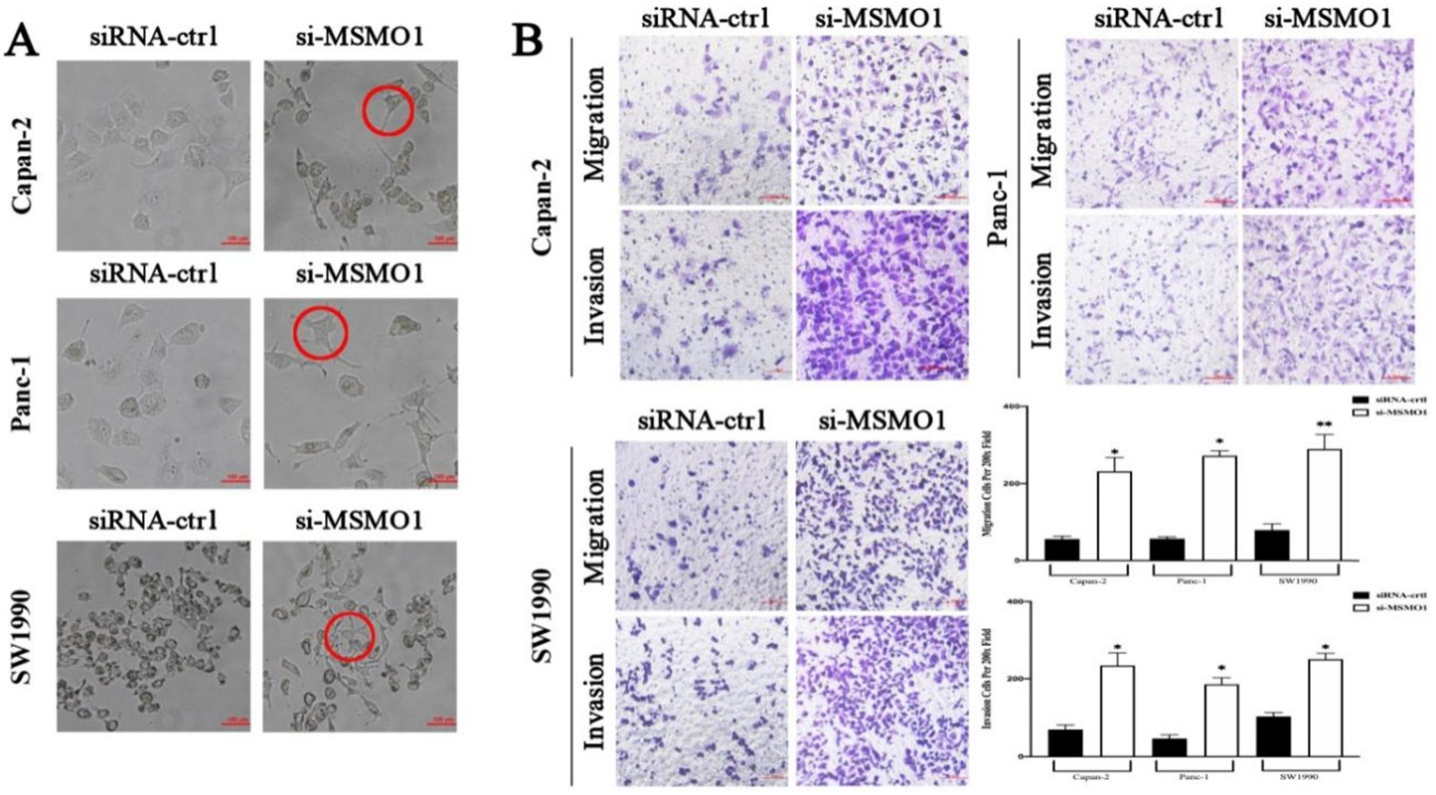
***In vitro*, MSMO1 silencing promoted PC cells EMT, migration and invasion. (A)** The EMT-like cell morphology of Capan-2, PANC-1 and SW1990 cells in MSMO1 siRNA and NC groups. **(B)** Migration and invasion in Capan-2, Panc-1, and SW1990 cells after MSMO1 silencing. Bars indicate means ± SD, *P < 0.05, **P < 0.01.

The essential key in cancer metastasis is EMT 15. To verify the effect of MSMO1 in PC cell function, we transfected RNA interference or negative control (NC) in Capan-2, Panc-1 and SW1990 cells. In results, the EMT-like cell morphology was stimulated by MSMO1 down-regulation, the cells lost their epithelial features and presented a spindle/fibroblast-like morphology, compared with the NC group, **(Figure 2A)**. As demonstrated in Transwell assays, the capacities of invasion and migration were significantly enhanced in MSMO1 siRNA transfected cells group in Capan-2, Panc-1 and SW1990 cells **(Figure 2B)**.

### MSMO1 Silencing Agitated EMT Activation via PI3K-AKT-MTOR Signal Pathway in PC Cells

**Figure 3:**
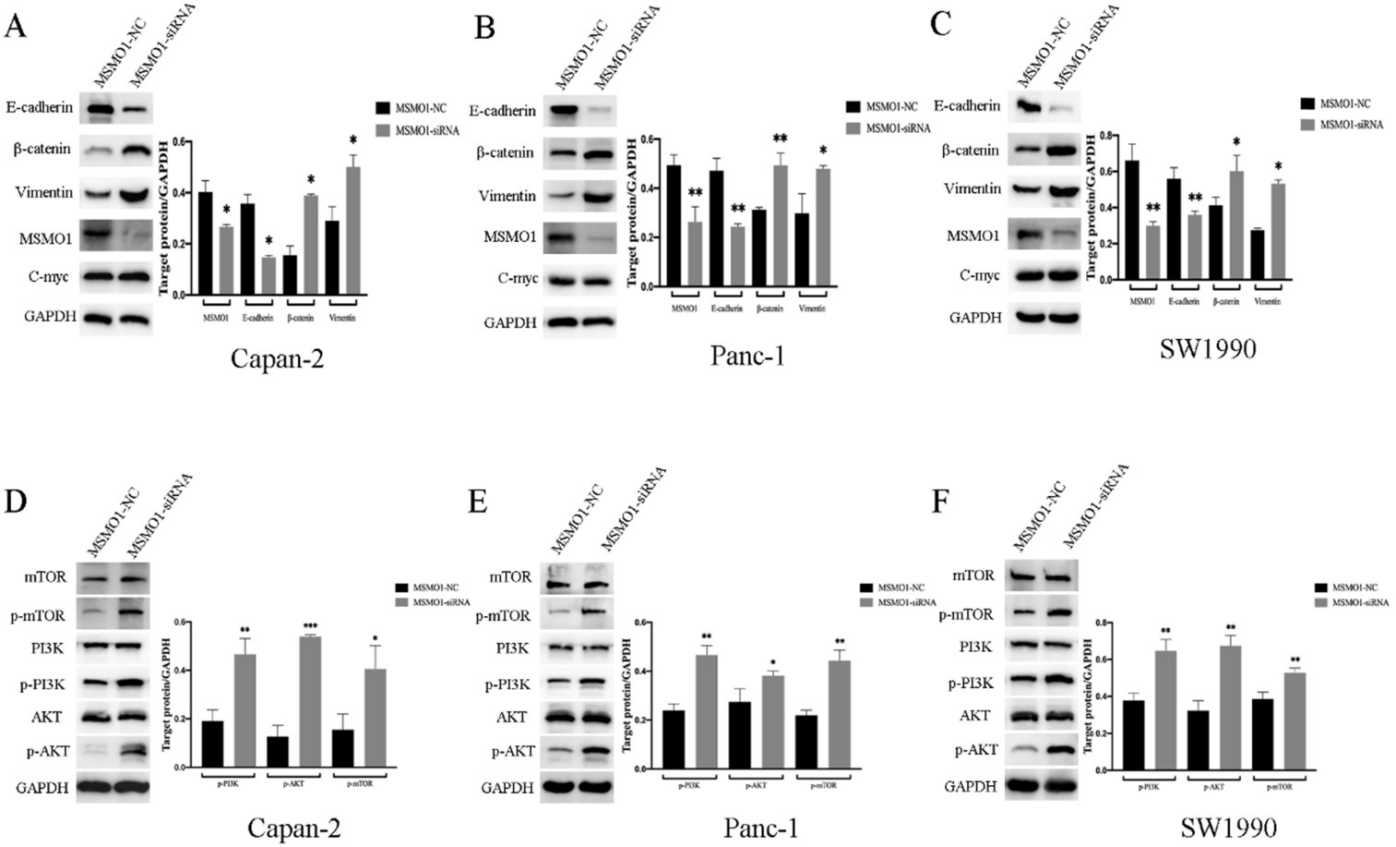
** Discrepancy of EMT-induced targets and PI3K-AKT-mTOR signal pathway *in vitro*. (A)** The protein level of MSMO1 and EMT features in Capna-2 cells. **(B)** The protein level of MSMO1 and EMT features in Panc-1 cells. **(C)** The protein level of MSMO1 and EMT features in SW1990 cells. **(D)** The changes of PI3K-AKT-mTOR signal pathway in Capan-2 cells. **(E)** The changes of PI3K-AKT-mTOR signal pathway in Panc-1 cells. **(F)** The changes of PI3K-AKT-mTOR signal pathway in SW1990 cells. Bars indicate means ± SD, *P < 0.05, **P < 0.01, ***P < 0.001.

For the purpose of exploring the potential mechanism of MSMO1 in PC cell function. Capan-2 cells, Panc-1 cells and SW1990 cells was used for MSMO1 silencing construction, and protein was then extracted from transfected cells to conduct Western blot. We found MSMO1 silencing inhibited EMT related protein (E-cadherin) and enhanced others (β-catenin and Vimentin). Some EMT markers showed no change, such as C-myc **(Figure 3A-C)**. Simultaneously, several proteins of PI3K-AKT-mTOR signal pathway [p-PI3K (Tyr458), p-AKT (Ser473) and p-mTOR (Ser2448)] was enhanced in MSMO1siRNA group **(Figure 3D-F)**. Based on the above results, the EMT induction and PI3K-AKT-mTOR signal pathway were both stimulated by MSMO1 silencing in PC cells *in vitro*.

### MSMO1 Down-Regulation Promotes Cell Proliferation of Pancreatic Tumors *in vivo*

**Figure 4:**
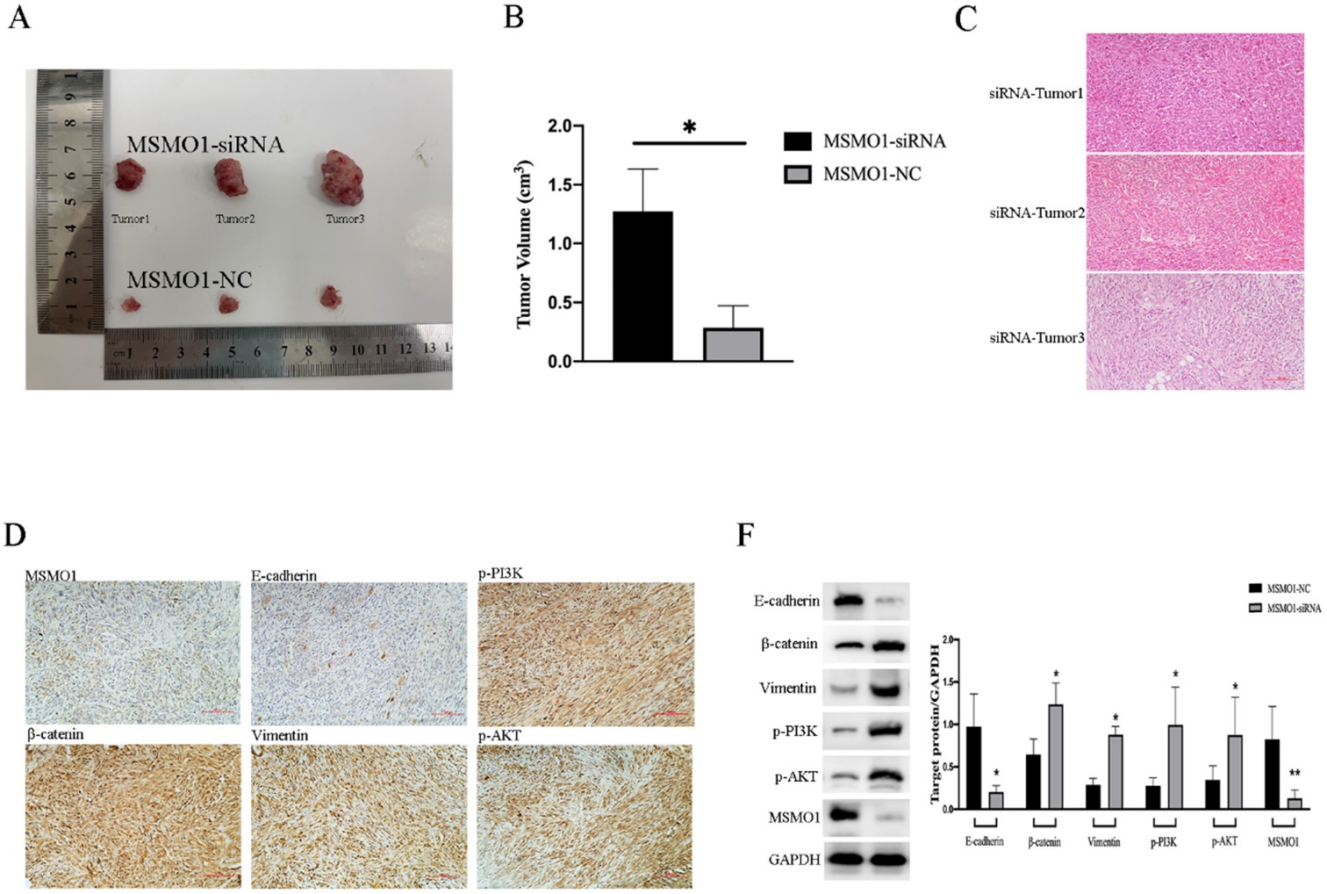
** MSMO1 silencing stimulated cell proliferation in subcutaneous tumor model *in vivo.* (A)** Subcutaneous tumor in MSMO1 siRNA and NC group. **(B)** The tumor volumes between MSMO1 siRNA and NC groups were evaluated by statistical analysis. **(C)** HE staining for subcutaneous tumor in MSMO1 siRNA group. **(D)** Target protein expressed in MSMO1 siRNA group detected by IHC. **(F)** The protein levels of MSMO1, E-cadherin, β-catenin Vimentin, p-PI3K and p-AKT in MSMO1 siRNA and NC transfected PANC02 cells detected by western blot. Bars indicate ± SD. *P<0.05, **P<0.01.

We constructed the models of subcutaneous tumor by PANC02 cells which were transfected with MSMO1 siRNA and negative control (NC), respectively. Tumor volumes in MSMO1 siRNA group were much larger (P=0.0138) **(Figure 4A, B)**. In histology, tumor tissues were observed by HE staining **(Figure 4C)**, besides, MSMO1 and E-cadherin expression were decreased but β-catenin, Vimentin, p-PI3K (Tyr458) and p-AKT (Ser473) expression were increased in Tumor was further confirmed by IHC **(Figure 4D)**. By western blot analysis, at protein level, Panc02 cell line exhibited E-cadherin expression was down-regulated, β-catenin, Vimentin, p-PI3K and p-AKT expression was up-regulated **(Figure 4F)**.

## Discussion

MSMO1, as a typical target in cholesterol biosynthesis, catalyzes the demethylation of C4-methylsterol in the cholesterol synthesis pathway and plays a key role in the normal synthesis of cholesterol 9, 16. Recently, MSMO1 is considered as a novel and critical gene target in various solid cancers, including breast cancer 11, oligodendroglia cells 12, hypopharyngeal cancer 17, gastric cancer 18, uveal melanoma 19, liver cancer 20 and cervical squamous cell carcinoma 21. Though the role of MSMO1 in PC radio-resistance has been reported in 2014, but the corresponding mechanism remains unclear 22. In current study, MSMO1 is identified as a novel tumor suppressor in PC development involving a novel signaling axis of EMT and PI3K-AKT-mTOR pathway, which was not reported previously to our knowledge.

Several researches have implicated the clinical significance of MSMO1 expression in various cancers. In breast cancer patients, targeting MSMO1 with siRNA increases the resistance to endocrine therapy 11. Down-regulation of MSMO1 was significantly associated with poor prognosis in liver cancer 20 and worse 5-years overall survival rates in gastric cancer 18. Currently, we first found that MSMO1 expression in normal pancreatic tissues was much higher than that in paired PC tissues. Meanwhile, low expression of MSMO1 was associated with advanced clinical stage (T stage, vascular permeation, lymphatic metastasis and poor prognosis) of PC patients, which is consistently with previous studies. The close relationship of low MSMO1 expression with the advanced clinical stage of PC patients drives us to investigate the potential role of MSOM1 in PC development *in vitro*.

EMT (Epithelial-mesenchymal transition) is considered as the initiating factor that converts benign carcinoma to the malignance with the high metastatic growth dissemination involving various signal pathways 23, 24. Our data implied a novel and specific role of MSMO1 in EMT here. EMT-like cell morphology and cell mobility were stimulated by MSMO1 silencing *in vitro*. Nowadays, the potential role of MSMO1 in cancer cells is still controversial. MSMO1 overexpression suppressed malignant cytological functional behavior in oligodendroglia cells 12. The high propensity for metastatic spread of uveal melanoma is the results of the transformation of MSMO1 19. Bioinformatics analysis presents the same trend in hypopharyngeal cancer 17 and cervical squamous cell carcinoma 21. However, MAGEA6 positively regulates MSMO1 and facilities the capacity of migration and invasion in esophageal cancer cells 25. Due to the different kinds of cancer and the related cellular environment, the function and mechanism of MSMO1 would be different and even contrary in various cancers.

Numerous evidences reveal E-cadherin is a critical EMT-related element 26. As previously, Numb-PRRL amplifies a set of EMT activators in PC, including E-cad and Vimentin 27. GATA6, the down-stream of Wnt-β-catenin signaling pathway, promotes EMT associated with E-cadherin downregulation 28. At the same time, we found MSMO1 down-regulation inhibited E-cadherin but enhanced β-catenin and Vimentin expression. To this context, the down-regulation of MSMO1 provided a potential unique point to influence EMT.

PI3K-AKT pathway plays a significant role in the malignant cellular function and the initiation of EMT 29. AKT regulates cell-cycle following the down-regulation of E-cadherin in EMT transmission 30, 31. mTOR, as the downstream target of PI3K-AKT signal pathway, also relates to regulate EMT^32^. Besides, AKT is the key down-stream effector of MSMO1 and inter-crossing protein between EGFR and PI3K/AKT/mTOR signaling pathway 33. However, MSMO1 has not been previously analyzed in the context of PI3K/AKT/mTOR signaling. In our study, WB shown that MSMO1 silencing increased p-PI3K (Tyr458), p-AKT (Ser473) and p-mTOR (Ser2448) expression *in vitro*. Thus, MSMO1 down-regulation facilitated EMT processing by mediating the PI3K/AKT/mTOR signaling pathway in PC, which is not reported yet. It is worthy noted that mTOR catalyze two multiprotein complexes, mTORC1 and mTORC2 34. mTORC1 promotes biosynthesis 35, while mTORC2 plays seminal functions in cell metabolism and survival through the p-AKT (Ser473) 36. In current study, we did not distinguish these two multiprotein complexes and the mTOR complexes will be a worthwhile point for us to explore in the future. Finally, MSMO1 down-regulation promoted the formulation of subcutaneous tumors accompanied with the promotion of EMT and PI3K/AKT/mTOR signaling pathway *in vivo*.

In conclusion, down-regulation of MSMO1 takes an important part in the aggressive clinical stage of PC patients. MSMO1 mediates EMT through activating PI3K-AKT-mTOR pathway *in vitro* and *in vivo*, which facilitates a novel diagnostic and therapeutic facility for PC. However, the corresponding mechanism is not further investigated in current study, which would be investigated in future.

## Figures and Tables

**Table 1:** The primer sequences

Gene name	Primer sequences
MSMO1	sense: 5' - TGCTTTGGTTGTGCAGTCAT‐3'; anti-sense: 5' - TTCCAAATGGAGCCTGAAAC-3'
GAPDH	sense: 5' - TGACTTCAACAGCGACACCCA-3'; anti-sense: 5' - CACCCTGTTGCTGTAGCCAAA-3'

**Table 2:** The Clinical Significance of MSMO1 Expression in Human PC Samples

Characteristics	No. of Patients	MSMO1 expression		χ^2^
		Low	High	
Case	92	49	43	
**Gender**				.910
Male	54	33	21	
Female	38	16	22	
**Age (y)**				1.000
≤65	23	12	11	
>65	69	37	32	
**Tumor location**				.817
Bead	66	36	30	
Body and tail	26	13	13	
**Tumor size (cm)**				.765
<4	79	43	36	
≥4	13	6	7	
**Differentiation**				.951
Well	30	16	14	
Moderate	57	30	27	
Poor	5	3	2	
**T stage**				.025*
T1+T2	64	29	35	
T3+T4	28	20	8	
**Lymph nodes metastasis**				.046*
Negative	63	29	34	
Positive	29	20	9	
**UICC staging**				.231
IA + IIA	69	34	35	
IIB + III + IV	23	15	8	
**Vascular permeation**				.009*
Negative	59	25	34	
Positive	23	23	9	
**Neural permeation**				.078
Negative	79	39	40	
Positive	13	10	3	
**Pretherapeutic CA19-9 level**				.291
<37 U/mL	54	26	28	
≥37 U/mL	38	23	15	

*P<0.05.

**Table 3:** The Univariate and Mutivariate Analysis of Clinicopathological factors For Survival

Characteristics	Median Survival (Days)	Univariate Analysis P (Log Rank)	Multivariate Analysis Hazard Ratio (95% CI)	*P*
Gender (Male /Female)	708/432	.910	-	
Age (≤65 />65y)	474/800	1.000	-	
Tumor location (Bead/Body and tail)	615/317	.817	-	
Tumor size (<4/≥4 cm)	629/321	.765	-	
Differentiation (Well/Moderate/Poor)	629/474/200	.951	-	
T stage (T1+T2/T3+T4)	800/317	.025	2.218 (1.120-4.393)	.022*
Lymph nodes metastasis (Negative/Positive)	660/418	.046	1.212 (0.637-2.305)	.588
UICC staging (IA + IIA/ IIB + III + IV)	708/275	.291	-	
Vascular permeation (Negative/Positive)	184/76	.009	1.223 (0.654-2.290)	.528
Neural permeation (Negative/Positive)	629/365	.078	-	
CA19-9 level (<37 U/mL/≥37 U/mL)	423//615	.291	-	
MSMO1 expression (low/high)	356/915	.001	0.431 (0.200-0.931)	.032*

*P<0.05.

## Abbreviations

MSMO1: Methylsterol monooxygenase 1
PC: Pancreatic cancer
EMT: Epithelial-mesenchymal transition
qRT-PCR: Quantitative real-time polymerase chain reaction
IHC: Immunohistochemistry
HE: Hematoxylin-Eosin Staining
RIPA: Radio Immunoprecipitation Assay
PMSF: Phenylmethylsulfonyl Fluoride
PVDF: Poly(vinylidene fluoride)
ECL: Enhanced Chemiluminescence
FBS: Fetal Bovine Serum
